# Media coverage, fake news, and the diffusion of xenophobic violence: A fine-grained county-level analysis of the geographic and temporal patterns of arson attacks during the German refugee crisis 2015–2017

**DOI:** 10.1371/journal.pone.0288645

**Published:** 2023-07-20

**Authors:** Thomas Hinz, Sandra Walzenbach, Johannes Laufer, Franziska Weeber

**Affiliations:** 1 Sociology Department, University of Konstanz, Konstanz, Germany; 2 AI Engineering, Schwarz IT KG, Neckarsulm, Germany; 3 Program and Funding, Stifterverband, Berlin, Germany; 4 ML Engineering, textada, University of Konstanz, Konstanz, Germany; Caleb University, NIGERIA

## Abstract

Over the year of 2015, about 800.000 refugees arrived in Germany, a number which equals around one percent of the total population. This migration process was labelled *the refugee crisis* and was accompanied by a contested debate. On the one hand, there was a widespread willingness to voluntarily help arriving refugees, on the other hand, the number of xenophobic attacks against refugees drastically increased. Our paper will focus on a specific form of xenophobic violence with a strong symbolic meaning: We analyze how arson attacks against collective accommodation facilities spread. Using a comprehensive web chronicle, we collected temporal and spatial data about arson attacks perpetrated on accommodations or facilities for refugees in Germany between 2015 and 2017. We counted 251 attacks, assigned each incident location to its county, merged county characteristics such as population size, proportion of foreigners, right-wing party support, and—going beyond previous research—added geographically coded media data from two digital archives. Besides newspaper contents of a popular nation-wide tabloid, we use a data base that covers local fake news on refugees. Based on these data, we constructed a balanced panel data set with the counties as geographical units and periods of 14 days as the time dimension. Results indicate that social contagion drives the diffusion process of arson attacks. Spatial proximity of previous attacks increased the propensity of attacks in the neighboring counties. Attacks were more likely to occur in counties with larger populations and fewer foreigners. While local newspaper coverage did not impact the diffusion of xenophobic attacks, fake news were relevant–but only in East Germany. We also considered two particularly salient threatening events that received nation-wide media attention, namely Merkel’s “border opening” on the 5^th^ of September 2015 and the sexual assaults occurring during New Year’s 2015/16 in Cologne. Both were followed by temporary increases in violence.

## Introduction

The migration of refugees from the Middle East to Europe has reached a dramatic peak in the fall of 2015. After an ongoing political and medial debate about the admittance and integration of refugees in Germany, Angela Merkel made the controversial decision to keep the border open for refugees on the Balkan route in the night from the fourth to the fifth of September 2015. Although strictly speaking, she decided to keep an already open border open, the night went down in history as Merkel’s “border opening”, a term that, for the sake of simplicity, we will also use throughout this paper. One popular German newspaper referred to the event as ‘the night Germany lost control’ [1]. In what followed, an unprecedented number of refugees arrived to Germany within a short period of time, adding up to a number of 800,000 people in 2015. If total population size is considered, this is less than some other European countries took in, however, the relative change from 2014 to 2015 multiplied. This influx of refugees was accompanied by two sorts of reactions from the German population: On the one hand, many volunteer groups formed to welcome and support refugees, e.g. by providing language courses or aid with bureaucratic issues. On the other hand, right-wing groups became increasingly visible in public demonstrations. Also violent actions against refugees reached a new maximum.

This paper focuses on a specific form of xenophobic violence with a strong symbolic meaning: We will analyze how *arson attacks* against refugee homes spread during the time period from the first quarter of 2015 to the first quarter of 2017, that is, a time span in which the immigration numbers rapidly rose, peaked and slowly declined back to more moderate levels after the Balkan route was closed in March 2016.

In order to understand the socio-geographic patterns and predictors of these xenophobic actions, we combine administrative data from the German Federal Statistical Office (Statistisches Bundesamt) and web-scraped data from different online sources. We applied a fine-grained geo-coding that allows us to geographically localize incidents and explanatory variables on a county-level.

Put more concretely, we built a rich data set that contains not only data on the location and time of 251 xenophobic attacks, but also socio-demographic information on the county (such as unemployment rates and the share of foreigners) as well as media coverage of previous xenophobic attacks and local fake news about refugees. Following the methodological approach proposed by Steele [2], we create an event data set and calculate a binary random effects model that enables us to examine how arson attacks diffuse through time and space.

One crucial question in this context is whether previous attacks increase the probability of further attacks. A common argument in the context of violence diffusion is that incidents are particularly likely to be picked up and discussed in the local media directly after an incident has happened, but for a very limited period of time (see e.g. [3,4]). Theoretically, we assume that a nearby attack and its media response can temporarily challenge social norms of humanity and tolerance, make violence against refugees more salient and seemingly more legitimate and should therefore increase the probability for future attacks in the area. Previous xenophobic attacks could hence create a temporary atmosphere of violence and a basis for the geographical dispersion of xenophobic arson attacks.

It seems likely that this trend is reinforced by a general atmosphere of mistrust and antipathy against refugees. This could be promoted by various factors: On the one hand, we will empirically assess if crucial historic events with a nation-wide impact were followed by temporary increases in violence, namely Merkel’s border opening in September 2015 and the sexual assaults committed predominantly by male refugees during New Year’s 2015/16 in front of the cathedral in Cologne. On the other hand, we will examine the share of extreme right-wing voters as a regional factor that is supposed to measure the general attitude towards refugees on the local level. In addition, we will capture regional differences with regard to two other factors: the share of foreigners and the local unemployment rates. These indicators refer to levels of perceived economic threat [5]. If these factors play a role, we would expect to see local differences in the distribution of violence across Germany, with a higher number of xenophobic attacks in counties with high unemployment and high immigration.

### Research questions

This paper is dedicated to the patterns of xenophobic attacks in Germany following the so-called *refugee crisis* in 2015/16. As mentioned, the high numbers of arriving refugees and asylum seekers were politicized far beyond right-wing extremist parties and led to unprecedented numbers of violent attacks against refugees and asylum seekers [for a historic overview, see 6]. We draw on many other studies that have investigated certain aspects of xenophobic violence against refugees in this time period [7–13]. However, our paper tries to include a wider picture of potential explanatory factors. Namely, we combine the substantive approaches of contagion effects, economic threat, contact theory, political opportunity structures, and look at the effect of media reports, fake news, and salient events that were perceived as threatening by the German population, namely Angela Merkel’s decision to keep the border open in September 2015 and New Year’s Eve in Cologne 2015/16. Summing up, we are interested in answering the following research questions:

- Do previous attacks increase the probability of further attacks in the area?
- Which roles do the media and fake news play?
- Do salient threatening events increase violence?
- Is violence a reaction to perceived economic threats?
- Do political opportunity structures of right-wing parties matter?
- Do opportunities for contact between ethnic groups diminish violence?

Our focus for this endeavor is on arson attacks (for the only other study that analyzes arson attacks separately, see [11]). Arson attacks have a long tradition in the history of xenophobia and racism [6], and are usually directed against the residential places where minority groups live or are designated to live. Arson attacks constitute a particularly visible and extreme form of violence with a clear signal: Offenders deny members of the minority groups the right to a new home. Due to the severity of this form of violence against refugees, such incidents should be less prone to underreporting than other common expressions of xenophobic violence, such as personal attacks or hostile demonstrations. In the following, we briefly introduce theoretical perspectives and related previous research on xenophobia.

### Contagion effects: Transmitting violence through the social network

The idea that behavioral phenomena spread through social networks like infectious diseases is not new. Just like medical or industrial innovations, fashion trends, drug use and social movements of all kinds [14–16], researchers have conceptualized political violence (such as wars, coups, disorders, terrorism, and homicides) as phenomena that diffuse through social networks by contagion [14,17–21]. In more recent years, scientific studies have increasingly focused on–and provided evidence for–contagion effects related to the spread of xenophobic violence in time and space [8,9,11,22,23].

In principal, contagion effects can refer to ideas as well as behaviors. In a nutshell, they can occur whenever information is transmitted form one actor to another within a social network. As Burt [24] puts it, social contagion requires an actor who already has adopted an innovation or a certain behavior, and another who has not. As soon as information is transmitted from the first to the second actor, an idea or behavior can be imitated and adopted, that is, contagion can occur. Two mechanisms have been put forward to explain the mechanics of contagion effects. Firstly, they can happen by personal contact and communication, usually by word-of-mouth. Secondly, it has been argued that physical proximity is not a necessary precondition for social contagion. Rather, Burt assumed that people are more likely to exchange information and imitate behavior if they are socially proximate or similar (“structurally equivalent” or “socially homogeneous”).

Applied to the field of xenophobic attacks, we expect a contagion effect that diffuses violence through time and space: After one attack has happened in a certain district, we expect a heightened risk for further attacks, within the same county and in adjacent districts due to geographical proximity.

In addition, we will test a range of socio-political characteristics on the county level that might affect the number of xenophobic attacks (such as population size, share of immigrants and unemployment rate). These will be discussed later in the context of other hypotheses. However, finding the same patterns of xenophobic violence in counties that are similar in these socio-political characteristics could be a hint towards a contagion effect that goes beyond geographical proximity (as suggested by Braun and Koopmans [22]).

### Media coverage

Social psychology offers an additional perspective on the spread of violence through contagion. If we assume that social norms are not fixed for all times but an agreement that we can renew or alter in our daily interactions (as symbolic interactionism would argue), different social norms can spread through a network just as any other idea and behavior. Some of these might be related to anti-immigrant sentiment, might downplay or trivialize violence and make xenophobic attacks more likely. This is why we now turn to the media, a powerful instrument of our time to disseminate ideas and change public opinion on a much bigger scale than personal communication.

We will distinguish between the following types of media contents:

- Reports on attacks against immigrants fueling contagion effects (might be prone to imitation, that is, contagion effects, possibly because they normalize violence)
- Anti-immigrant sentiment in social media and fake news (seem particularly suited to evoke changes in social norms)
- Salient threatening events with omnipresent media coverage such as Merkel’s border opening and New Year’s 15/16 (might influence public opinion in a way that justifies violent behavior against foreigners)

### Media reports on attacks against immigrants driving contagion effects

Media reports on arson attacks can be better understood from the perspective of agenda setting and framing theory [25–27]. Media outlets have the power to determine which aspects of the refugee crisis receive attention and how they are portrayed to the public. They can choose to highlight certain events, individuals, or perspectives while downplaying or ignoring others. These processes of agenda setting can be driven by different motivations including political affiliations, commercial interests, or the desire to cater to target audiences. They can shape the public’s perception of refugees and might potentially foster anti-immigrant sentiment.

On the one hand, agenda setting involves topic selection, in this case the decision whether to cover the refugee crisis as a significant news item at all, on the other hand it refers to the narratives that are conveyed with the media report. How is the topic framed: Does the report focus on an individual human story, on economic impacts, security concerns or political dimensions? Who is given a voice: politicians, NGOs, or refugees themselves? Which emotions are evoked by visual representations?

In the context of xenophobic violence, where the media is a powerful tool of transmitting violent behaviors through networks, many studies have focused on which events are reported and how prominently this is done. The mere number of studies on media portrayals of refugees in different European countries shows how important the media are and how controversial their role is [28–32].

Some of these analyses examine contents, some focus on the selection of contents. To name some non-representative examples, Georgiou and Zaborowski [33] conduct a content analysis of media reports related to the refugee crisis in eight European countries and conclude that immigrants were often either portrayed as vulnerable or as dangerous outsiders. Over time, empathetic reports were increasingly replaced by narratives of suspicion. Concerning media coverage, some researchers talk about selection bias, suggesting that attacks against immigrants that involve more victims and take place in closer proximity are more likely to be reported [3]. Others argue that key events trigger an overrepresentation in media coverage for following similar events [4] or point towards the disproportionate media coverage of dramatic, rare and violent content [34].

The worry is that such news reports trigger a temporary shift in social norms, making xenophobic violence more legitimate for others. This is in line with broken window theory, which argues that visible deviant behavior has a signaling function for other actors who are led to believe that such behaviors are accepted [35]. Dependent on the tone of the report, it seems plausible that media coverage of xenophobic events can have the power to challenge social norms of tolerance, human rights, and peaceful coexistence in favor of violence.‬‬‬‬‬‬‬‬‬‬‬‬‬‬‬‬‬‬‬‬‬‬‬‬‬‬‬‬‬‬‬‬‬‬‬‬‬‬‬‬‬‬‬‬‬‬‬‬‬‬‬‬‬‬‬‬‬‬‬‬‬‬‬‬‬‬‬‬‬

Empirically, some studies have found links between media coverage and outbursts of violence [22,23,36]. For example, Braun & Koopmans [22] find that the rate of violent events against immigrants increases when the media previously covers such events in a more salient way (that is including a photo or reporting on the front page). Both, Braun and Koopmans [22] and Jäckle and König [9], found that the number of negative statements about immigrants in the media increased violence in the subsequent time period. Braun [23] does not replicate this effect but finds that any the mere salience of immigration as a topic on the news increases violence, irrespective of tone. In addition, media reports of previous attacks against immigrants made further attacks more likely.

There also is a scientific consensus that the effects of media coverage on xenophobic violence are rather short-lived, that is, they mark an only temporary change in social norms. In his study on racial rioting in the US, Myers [3] finds that severe riots increase the chances for further outbreaks for about one week before the effect diminishes. Braun [23] uses a time period of the previous 30 days to measure diffusion of xenophobic attacks in the Netherlands.

### Fake news fostering anti-immigrant sentiment

Apart from media reports on xenophobic attacks, we argue that also fake news play a crucial role in spreading anti-immigrant sentiment. Previous research suggests that right-wing organizations typically use social media to deliberately spread misinformation and hatred [37]. Wahlström and Törnberg [38] identify three mechanisms by which social media can generally lead to violence: a) users look for information that confirms their beliefs, b) they express mutual recognition and legitimization of extreme beliefs, and c) they share practical information (e.g., on how to burn down a house) and co-ordinate violent activities.

Müller and Schwarz [39] empirically show that anti-refugee sentiment on Facebook predicts crime against refugees on a municipal level in Germany by investigating users that engage on the AfD (Alternative für Deutschland, right-wing populist party) facebook page. An additional characteristic of social media that seems crucial is its lack of content management or supervision. Since anyone can post and share contents without any serious quality control, extreme perspectives can easily be expressed. These circumstances make it particularly easy for more and less organized right-wing and populist entities (for an overview see e.g. [40]) to actively engage in spreading their ideology and influencing public opinion, while fact-checkers are facing difficulties in disseminating their corrections [41].

To the best of our knowledge, no other study has examined the correlation between fake news and xenophobic violence during the refugee crisis. However, there is some descriptive research on fake news in other contexts, such as the Myanmar genocide [42], the mob lynchings in India [43], the storming of the US capitol [44], and the Brazilian Presidential Election 2018 [45]. A study on the presidential election 2016 in the US [46] shows that social media users are much more likely to believe fake news that corroborate their own ideological and political beliefs. From a psychological perspective, this finding relates to concepts of motivated reasoning and confirmation bias, suggesting that people are more likely to consume and accept information that reinforces their already existing beliefs, while perceiving information that contradicts their views as less convincing [47]. For violence against refugees, this means that exposure to fake news is unlikely to convince people with opposed opinions but might well contribute to the radicalization of people with pre-existing anti-immigrant sentiments. Such processes of radicalization seem particularly concerning, since we know from previous research that fake news spread faster through social media than facts [48] and that negative emotions can easily become contagious to other people in an online setting [49].

### Salient ‘threatening events’ changing public opinion

Slightly different from Jäckle and König [9], who focus on terrorist attacks and their impact on xenophobic violence, we use the term ‘threatening event’ for the rare incidents when media reports that portrayed refugees as a threat became omnipresent throughout the whole country. The most obvious example of such an incident surely is New Year’s Eve in Cologne 2015/16, during which women were insulted, sexually harassed and occasionally robbed, mainly by refugees from Algeria, Morocco, Iraq and Syria. The event was omnipresent in the German media for a while, particularly because local police seemed to be unable to cope with the situation. It is a wide-spread belief that the tone of news reports and the public opinion towards immigration changed as a consequence of these events, particularly towards male Muslim refugees [32,50]. Köttig and Sigl [51] argue that the night "marked a turning point in perceptions of the need to protect refugees, especially male refugees". This result is somewhat confirmed in a factorial survey that finds lower acceptance towards immigrants after New Year’s Eve 2015 [52], although the experimental design of the study is not flawless [53]. In addition, some studies find that the incidents at New Year’s Eve 2015/16 were indeed followed by a rise in xenophobic violence against refugees [10], possibly as an act of revenge against a perceived out-group. A similar shift seems to have happened after Angela Merkel decided to keep the borders open for refugees on the Balkan route in September 2015 (Merkel’s ‘border opening’). The period that followed has been coined the ‘refugee crisis’.

Unlike xenophobic attacks, threatening events do not offer a clear behavior that could be imitated. Instead, they are connected to a more or less vague compilation of anti-immigrant sentiments, which can contribute to a shift in public opinion and a temporary or permanent change in the acceptance of violence against immigrants. *We expect direct nation-wide effects on xenophobic violence*, going beyond attacks in geographical or social proximity to previous attacks.

### Economic threat

Although the impacts of immigration on wages and employment seem to be rather small (for a meta-analysis see [54]), a popular argument when talking about anti-immigrant sentiments and xenophobic violence is the concept of economic threat or deprivation [55]. The idea is that competition for scarce goods, such as employment, is what triggers xenophobic attitudes and actions. In other words, *xenophobic attacks should be more likely where unemployment is high and numbers of immigrants are large*.

Generally speaking, empirical evidence on the topic is mixed [56,57]. Studies range from analyses of survey data on attitudes about immigration [58–61] or ethnic prejudice [62] to macro-level analyses of the correlation between economic threat and interracial crimes in the US [63,64] or economic threat and right-wing violence in Germany [65,66].

Out of the studies that have investigated xenophobic violence during the refugee crisis in Germany, none has found convincing empirical evidence for this idea. Generally, studies do not find an effect of unemployment rate or other related measures [8,9,11,13]. In their study on anti-immigrant attitudes, Entorf and Lange [7] report a small effect that disappears when controlling for East- versus West-Germany.

One obvious reason for this apparent lack of economic threat is that refugees face serious disadvantages on the German labor market. Their right to work depends on their residence status, and their capacity to compete is limited by potential problems to get their degrees recognized. Nonetheless, some researchers have argued that xenophobic attitudes might be triggered by subjective perceptions of economic threat rather than objective situations of greater competition. This idea is somewhat supported empirically by Semyonov et al. [67] who find that the perceived size of the foreign population is associated with perceived threat while the actual size is not.

### Contact theory

A somewhat contradictory effect of immigration would be expected in the light of contact theory [68]. In a nutshell, it states that contacts between different ethnic groups reduce prejudice. *Higher numbers of immigrants* yield more opportunities for contact between the majority and minority groups within a society and *should prevent or reduce prejudice on both sides*.

Empirical evidence is somewhat mixed. Among the studies that have analyzed xenophobic violence in the context of the refugee crisis in Germany, some have found support [7–9] or partial support [11,13] for contact theory. Schaub et al. [12], however, who examine refugee-related attitudes and behaviors around the same time, come to a more skeptical conclusion. The study uses a matching approach to compare survey data from 236 German municipalities, out of which half received refugees, while the other half did not. Interestingly, the authors find that local exposure to refugees did not have any effects on right-wing, populist or anti-refugee attitudes. Similarly, the following natural experiment on attitudes in the context of the refugee crisis comes to skeptical conclusions: Using survey data from different islands in the Aegean Sea that have partly experienced considerable refugee arrivals while others have not, Hangartner et al. [69] find sizable increases in hostile attitudes against refugees and immigrants among the local population.

More generally speaking, the idea that contact reduces prejudice has been corroborated in some studies [70,71], while others have found the opposite effect [22, see 72 for an experimental trial]. Two meta-analyses, one of them only on studies with random assignment to treatment groups, come to the conclusion that contact “typically reduces prejudice” [73,74].

Wagner points out a reason why some studies might fail to find empirical support for contact theory. He argues that mere contacts are not enough: “It is friendship, that is, intimate and personal contact, which has an effect on prejudice.” [27,75]

Piatkowska [76] offers an alternative interpretation for the negative effect of immigration on xenophobic violence that she finds: As the size of the minority population increases, their potential power and the possibility for retaliation increases and therefore makes crimes against them less likely (“power-differential hypothesis”).

### Prevalence of right-wing/populist parties and ideology

As previous research has argued, right-wing parties portray immigrants as enemies, evoke feelings of hatred and, in this way, contribute to a political climate that is more conducive to xenophobic violence [77]. Particularly PEGIDA and later the right-populist AfD became popular actors during the refugee crisis for fueling the fear that Germany would be swamped by uncontrolled mass immigration, bringing chaos and terrorism to the country [78,79]. In addition, right-wing and populist parties are usually well connected to other groups that are susceptible to xenophobic violence and easy to mobilize [80].

In line with typical right-wing ideology, frequent motives among perpetrators are competition for jobs, cultural or value-related threat, exploitation of the German security system ([81], although the author also points out that other non-ideological motives such as group dynamics might drive xenophobic attacks). In light of these considerations, *one would expect that greater strength of right-wing parties and hence visibility of right-wing ideology should shape public opinion in a way that fosters xenophobic violence*.

Interestingly, also the exactly contradictory argument has been put forward in previous research on xenophobic violence [22,82]. The underlying idea is that representation within the established circles of politics channels anti-immigrant sentiments into legal boundaries and makes extra-institutional mobilization superfluous. In the context of xenophobic violence, this means that *a strong representation of right-wing parties on the municipal level should reduce the need for extra-institutional violence against immigrants*. Looking at previous research, empirical evidence that the strength of right-wing parties inhibits extra-institutional outbursts of xenophobic violence is provided by some studies [22,83], but not by others [23,82].

### East vs. West Germany

Many studies on xenophobic violence in Germany test and find differences between the West and the former Socialist East, with the East yielding consistently more xenophobic violence despite far lower numbers of immigrants (see e.g. [8]). Sometimes these differences can be partly or entirely explained by unemployment levels or other socioeconomic characteristics, sometimes they remain significant despite controlling for such variables [13,22,65,84]. Adam [85] sees the reason for the remaining socio-cultural differences in the “official denial of the fascist legacy” in East Germany, while the West engaged in an “effective re-education policy” (p.1), for an explanatory attempt on the basis of survey data, see [86]). Whatever the exact reasons might be, we will distinguish between East and West Germany to capture such socio-cultural differences.

Summing up, we hypothesize that the following indicators correlate with the likelihood of xenophobic attacks. If not stated otherwise, we expect positive correlations.

(a) the number of previous attacks in spatial neighborhoods
(b) local media reports on attacks nearby
(c) anti-immigrant fake news originating nearby
(d) salient threatening events with nation-wide media-coverage

(indicated by a dichotomous variable that marks the time point of these events)

(e) unemployment in the county
(f) share of immigrants in the county

(contact theory predicts positive, economic threat literature negative effect)

(g) right-wing voters in the county
(h) East (vs. West) Germany

## Data and methods

We build on a combination of different data sources characterizing 402 counties in Germany: official statistics, data from web sources, and compiled and pre-processed media information. For each data source, collection and analyses comply with the respective terms and conditions, and we will use this section to provide further details on all indicators used.

To construct a reliable dependent variable that measures violence against refugees, we collect temporal and spatial data about *arson attacks*. We focus on arson attacks as a highly visible and aggressive form of violence. Even if arson attacks are primarily directed against facilities, offenders consciously risk that individuals get seriously hurt or die. Methodologically, this means that arson attacks are less likely to be underreported than other types of xenophobic violence.

We gather information about arson attacks perpetrated between year 2015 (first quarter) and 2017 (first quarter) on accommodations or facilities for refugees in Germany from the web chronicle *Chronik flüchtlingsfeindlicher Vorfälle* (https://www.mut-gegen-rechte-gewalt.de/service/chronik-vorfaelle). The data are provided by the Amadeu Antonio Foundation and PRO ASYL, and do not only include arson attacks on inhabited refugee accommodations but also on facilities under construction. In addition, it includes all kinds of facilities provided by local authorities to accommodate refugees: larger buildings for mass accommodation but also smaller units such as apartments, family houses, etc. Data basis of the chronicle are publicly available newspaper reports, press releases of the police as well as reports from local and regional helpdesks for victims of right-wing, racist, and antisemitic violence [87]. The same data source was used by other researchers [8–11], although most of them do not distinguish between arson attacks and other forms of violence.

**Fig 1:**
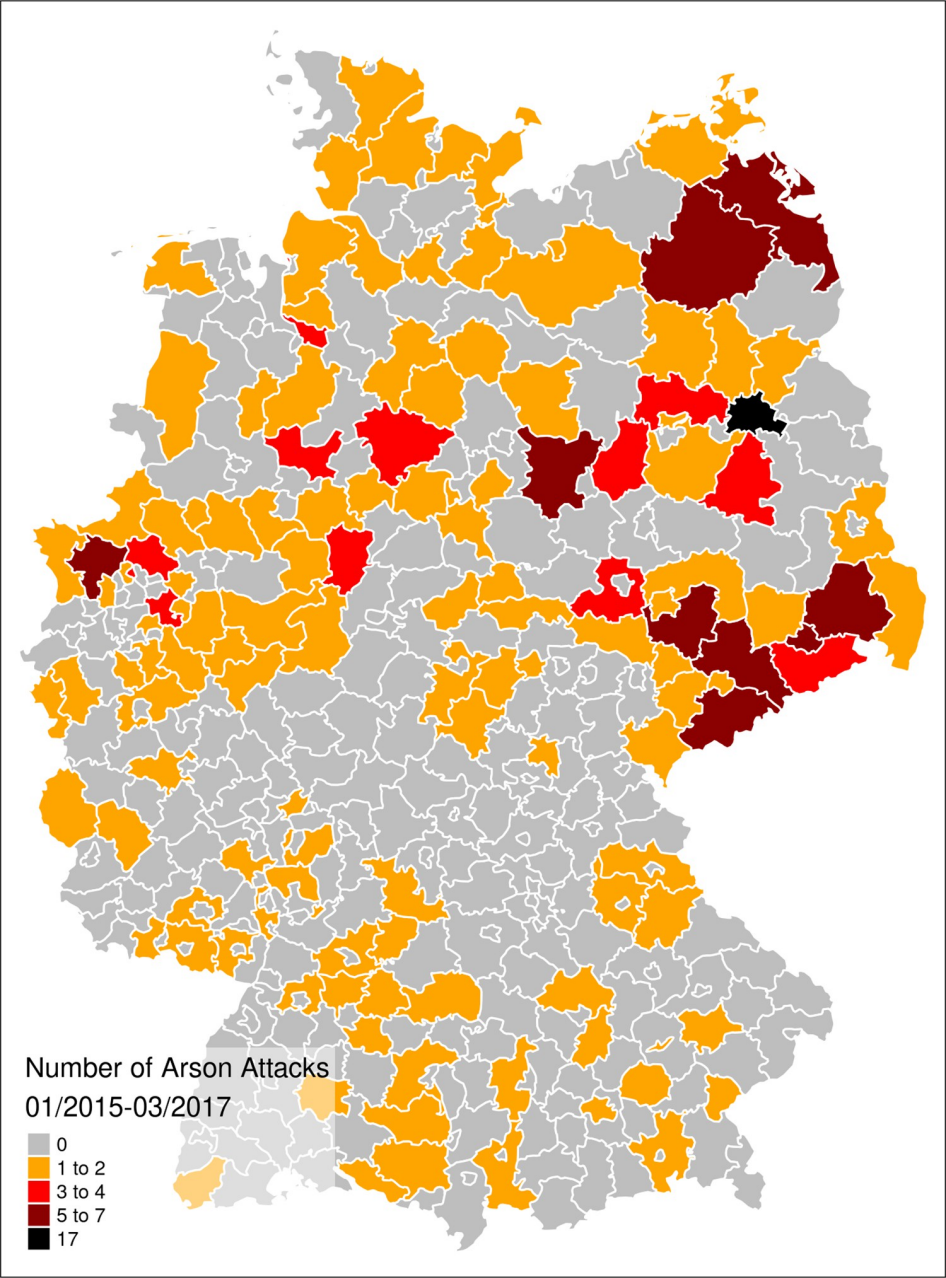
Number of arson attacks on refugee facilities in Germany (1/2015–1/2017); own illustration based on *Chronik flüchtlingsfeindlicher Vorfälle*.

For our observation window, we count 251 arson attacks in total, with a regional focus in East Germany (see Fig 1). Although only 15% of Germany’s population lives in the East, 44.2% (111) of our registered arson attacks take place in this region.

Using Google’s Geocoding API, we assign each incident location to its respective county. We arrange the event data as a balanced panel data set with the county identifier as the individual dimension *c*, and a period of 14 days as the time dimension *t*. This leaves us with 59 time intervals of two weeks (from 2015 to 2017) and 23,718 county-periods for analysis. The resulting binary arson attack variable for a county-period *ct* is equal to 1 if one or more incidents occurred in that specific county-period. This gives us a dependent indicator variable with 227 counties experiencing at least one attack in the observation time, 43.2% (98) of them in East Germany. Subsequently, we will describe how we further enriched this panel with time-constant and time-variant county-level data, and with time-variant nation data.

### Time-variant county-level data (lagged variables)

To test hypothesis a, we generate a *lag variable indicating (previous) attacks* in the time period before the current time period for all counties and their (direct) neighboring counties. We use this next neighbor approach (all neighbor counties with a common border to the county of focus) for the main analysis.

As mentioned earlier, we categorize the time from 2015 (first quarter) to 2017 (first quarter) into 59 intervals of two-weeks each. As a consequence, the actual time interval between two different attacks measured by the indicator variable in one county could be between one day and almost four weeks. The number of (previous) attacks ranges from 0 (in 93.6% of all counties in the space-time matrix) to 6 (for only 10 counties in the space-time matrix). For 1,482 counties, at least one attack occurred in this or a neighboring county in the previous time period. Note that using the lag variable reduces the number of cases by 402 since there is no lagged information available for the first time interval under study. This leaves us with a total of 23,316 county-periods for analysis.

### Local media

As outlined above, we additionally consider the local media coverage of (previous) attacks (hypothesis b) as well as fake news about refugees (hypothesis c) with a geographic reference. Equivalent to the specification used for previous attacks, we model their delayed and their spatial impact.

We approximated the media coverage of the event data with the help of the DIGAS press database provided by the Axel Springer Syndication. We decided to use the regional editions of BILD. BILD is a very popular daily tabloid newspaper that is read all over the country. It is particularly suited for our purposes because it does not consist of just one nation-wide edition but instead comes with a few pages on regional news with a more targeted geographical focus, which is where we would expect coverage of local arson attacks. These regional editions are comparable in scope and provide consistent coverage across the country. A list of the included regional editions of BILD can be found in S2 Appendix. For each arson attack, we restricted the text corpus to reports published on the incident date up to one week after the incident. We searched with the following (German) search query:

(Brandanschlag OR Brandsatz) AND (Asylbewerberheim OR Flüchtlingsheim OR Asylbewerberunterkunft OR Flüchtlingsunterkunft OR Asylunterkunft OR Asylheim)AND(`Incident Location´)

It combines the keyword “arson” with a range of terms referring to facilities for refugees. `Incident Location´ reflects the location string of the current incident.

Before querying, we removed over-identifying words from the location string, meaning that e.g. Berlin-Kreuzberg was reduced to Berlin. After removing false-positive hits from the result lists, we generated a binary variable indicating whether an arson attack was covered by the newspaper or not. Transferred to a panel lag format and using the neighbor concept for counties, the media coverage variable reflects the number of arson attacks reported on in a specific county-period *c*_*t-1*_ for the county and all direct neighbours.

In total, this strategy leads to a total number of 265 media coverage units in BILD. In 98.9% of all counties in the space-time matrix, we record no media reports. As expected according to hypothesis b, local media coverage correlates with the dependent variable of arson attacks (.377; p < .001). In addition, there was no media coverage without a registered event in the chronicle, which highlights the reliability of the data.

### Fake news

We use spatial event data from the hoax map project (https://hoaxmap.org/) to calculate the *number of fake news* spread in a county-period *c*_*t-1*_. The website lists discrediting information about refugees that proved to be erroneous. In addition to rumors about refugees, rumors about people with southern complexions or non-German accents are included in the data base because such characterizations are typically associated with refugees. The most frequent accusations concern refugees robbing, assaulting other men and sexually harassing women.

For each piece of fake news, the database contains a location string of the supposed incident, which we assign to its respective county with Google’s geocoding API. The total number of fake news gathered is 416 (132 in East Germany, 31.7%). After transferring the original information to the time-space matrix (considering neighbor counties and the previous time period), there is again huge variation over time and space: 92.1% of units do not record any fake news. The maximum for one unit of analysis was 29 pieces of fake news (Kreis Lippe, West Germany). In sum, we counted 2.759 pieces of fake news in the time-space matrix (neighbor counties included). This refers to 1,837 county-periods with values greater than or equal to one.

For the computation of neighbor variables, we used a county-level geography of Germany provided by the *Bundesamt für Kartographie und Geodäsie* (territorial state: 01.01.2016). As mentioned briefly above, for each county, we identified all counties that have a common border with the county by means of the R-package spdep. Then, we computed the sum of arson attacks, whether an arson attack was covered by Bild, and the sum of fake news assigned to the county and the neighboring counties for a county-period *c*_*t-1*._

### Time-variant nation data

#### Salient threatening events

Concerning hypothesis d, we trace the influence of salient threatening events, namely Merkel’s border opening in September 2015 and the New Year’s Eve incidents in Cologne 2015/16). For this, we generate two indicator variables that mark the subsequent four time periods (i.e., two months) after the period of the respective event. We gave the events a decay function that expresses the shrinking impact of an event as time goes by [88].

#### Time-constant county-level data

The German Federal Statistical Office (Statistisches Bundesamt) provides county-level information via the DESTATIS database on the *unemployment rate* (2015), the *proportion of foreigners* (2015), *the proportion of asylum seekers* (2015) and the *number of inhabitants* (2015). The county-level *share of NPD voters* in the last general election (2013) was retrieved from the webpage of the Bundeswahlleiter.

*Unemployment Rate*. The unemployment rate in a county is a proxy variable for the economic threat people might associate with immigrants (hypothesis e). On average, the unemployment rate is 6.7%, with a range from 1.5% (Eichstätt, West Germany) to 16.8% (Bremerhaven, West Germany).

#### Foreigners

The *proportion of foreigners* in 2015 is used as an indicator for previous exposure to immigrants (hypothesis f). With regard to the theoretical concepts, the variable serves two purposes: First, it is a proxy for the contact probabilities between the general population and the non-native population within the counties. Secondly, the proportion of foreigners potentially also signals economic threat. To better distinguish these mechanisms empirically, we introduce an *interaction term* between the proportion of foreigners and the unemployment rate in the counties. If economic threat is the mechanism that matters, we would expect xenophobic violence to be highest were immigration numbers and unemployment rates are both high.

The proportions of foreigners per county range from 1.9% (Elbe Elster Kreis, East Germany) to 33.6% (Offenbach, West Germany). There is clearly a huge difference in immigration by region: On average, 3.9% of the population in East German counties and 10.1% of West German counties are non-Germans).

#### Right-wing voters

The county-level share of NPD voters is used as a measure of the prevalence of extremist right-wing ideology (hypothesis g). We used data from the general election of 2013 and look at voter turnout for the 402 counties separately. The overall average of NPD votes is 1.4%, with a range from 0.2% (Münster, West Germany) to 5.1% (Sächsische Schweiz Osterzgebirge, East Germany). Again, there are remarkable differences between East and West Germany. Within the quintile of counties with the highest NPD vote share, 85% of counties belong to East Germany.

#### Population

In our empirical analysis, we use population size as a control variable since crime rates are generally higher in more populated areas. (For this, we divide the original counts by 100,000). The variable is skewed with 75% of counties being smaller than 240,000 inhabitants and only 5% of counties having more than 500,000 inhabitants.

Looking at the correlation matrix of all time-constant county-level variables (see S1 Appendix), there are only two correlation coefficients with values above .5: The East-West dummy variable (1 indicating counties in East Germany) correlates positively with the share of NPD voters (.813; p < .001) and negatively with the proportion of foreigners (-.505; p < .001), suggesting higher levels of anti-immigrant sentiments despite lower levels of immigration. In line with theoretic considerations, these bivariate results give us reasons to examine the patterns of xenophobic violence for East and West Germany separately, in addition to a model that covers Germany as a whole.

### Statistical model

Our analysis strategy is inspired by Steele’s application of a two-level binary random intercept model to discrete-time event history data [2]. In contrast to traditional event data analysis, this means that a county in our data is not only analyzed until a xenophobic attack happens, but several attacks might happen over time. At the same time, we acknowledge the multilevel structure of the data, where multiple time periods (level 1) are observed for each county (level 2). Using the *xtlogit* command in *stata*, we estimate the log-odds of an arson attack in a given county *c* at time *t* as follows:

$$ln\left(\frac{P{(Y}_{ct}=1)}{P{(Y}_{ct}=0)}\right)=\beta_{0}+\beta_{1}W_{c}{+\beta }_{1}X_{t}+\beta_{2}Z_{c1(t-1)}+\cdots +u_{0c}+e_{0ct}$$

where *β*_0_ is the mean intercept, *u*_*c*_ represents the random effect on the county level and *e*_*ct*_ the idiosyncratic error, which are both assumed to follow a normal distribution. As predictors, the formula includes *W*_*c*_, a vector of time-constant county-level variables (e.g. unemployment rate 2015), *X*_*t*_ for time-variant nation data (e.g. New Year’s Eve with decay function), and *Z*_*c*1(*t*−1)_, lagged time-variant county-level variables (e.g. arson attacks in same or neighboring counties for the previous time period, an indicator that measures if the risk of an attack increases if the same county or an adjacent one has experienced an attack in the previous time period).

To facilitate interpretation, we will report average marginal effects (AME) instead of logit coefficients throughout this paper. This means that the coefficients indicate the average changes in the probability of an arson attack if the predictor variable increases by one unit.

## Results

The prevalence rate for all 402 German counties to experience an arson attack over the observation time from 2015 until 2017 is at 0.96 percent (227 out of 23,716 units). As Fig 1 already illustrated, East Germany has a higher prevalence value (2.16; 98 out of 4,543 units) than West Germany (.67; 129 out of 19,175).

We follow a straightforward multivariate strategy to detect the spatial and temporal patterns of arson attacks.

**Fig 2:**
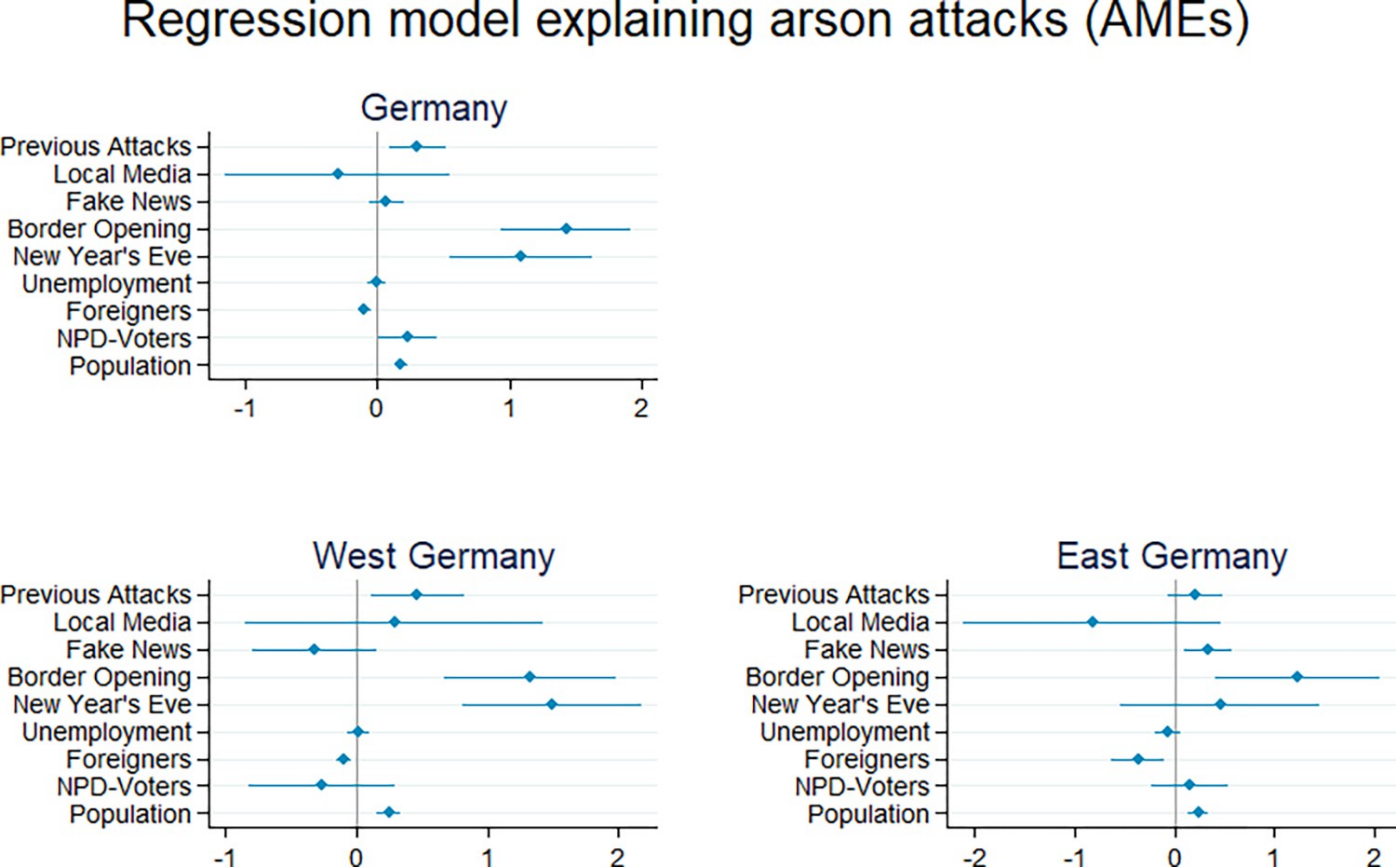
Average marginal effects, point estimates with 95% confidence intervals.

Fig 2 shows the average marginal effects from the discrete event history model predicting xenophobic arson attacks. All the indicators we discussed above were included as explanatory variables. The first graph in Fig 2 shows the estimations for the complete data set, covering Germany as a whole, and second and third graph refer to East and West Germany respectively.

The three last variables in each model refer to the spatial *and* temporal processes of social contagion and media influence. For the total data set of German counties, the number of *previous attacks* (in the county and neighboring counties) increases the likelihood of a further arson attack by around .3 percentage points–i.e., on average the prevalence raises from .96 to 1.26% per registered previous case. This result is statistically significant at the 1% level (*p* = .005) and in line with our expectation about the spatial and temporal proximity for the diffusion of violence. In West Germany, the spatial and temporal dependency is a bit more pronounced and also statistically significant (*p* = .010), while in East Germany the slightly smaller point estimate misses the 5% threshold of statistical significance (*p* = .147). We interpret these results as at least partial support for hypothesis a.

In contradiction to hypothesis b, the *local media coverage* of attacks does not impact the process of diffusion. The estimates for the complete data set and both parts of Germany have large confidence intervals, meaning that there is no correlation with the measure of local media coverage and the attack diffusion.

We also checked whether the coefficient for this variable becomes stronger when previous attacks are not accounted for (see models 2 in S3 Appendix). We found no evidence for local media impact in this restricted model. However, this does not imply that media coverage is not relevant at all. Note that our variable measures a very specific form of *local* media coverage, the 15 regional editions of a nationwide tabloid.

*Fake news* about refugees with a local reference are in tendency positively correlated with arson attacks. In the joint model for East and West Germany, the average marginal effect is very small (.071) and does not reach statistical significance (p = .301). Similar results were obtained in models without previous attacks and local media coverage (see model 3 in S3 Appendix). However, separate analysis by region reveals an interesting pattern: In East Germany, fake news seem to drive the process more clearly (effect size: .329 p = .006) while in West Germany, the point estimate even has a negative sign (effect size: -0.321; p = .182). Substantively, we conclude from the estimation that fake news on social media mainly impact the spatial and temporal patterns of xenophobic violence in 2 East Germany, where people show limited trust in politicians [89] and the mainstream media [90]. Hypothesis c is supported for Eastern Germany.

Looking at the two salient threatening events with nation-wide media coverage that occurred in our observation time, the analyses show that both, border opening and New Year’s Eve, remarkably impact the overall pattern (hypothesis d). Shortly after the decision of the German government to keep the *border open* for refugees who made it on the Balkan route to Hungary (in the beginning of September 2015), the arson attacks experienced a peak. The significant coefficients in Fig 2 shows that the border opening drives the likelihood of arson attacks upwards by 1.434 percentage points (*p* = < .001), with very similar patterns in East and West Germany (East: 1.236 with *p* = .003; West: 1.327 with *p* < .001).

Overall, New Year’s Eve seems to have a smaller effect. For Germany as a whole, the respective dummy variable is highly significant (1.094 percentage points; *p* < .001). In West Germany, the period effect of New Year’s Eve is highly significant (average marginal effect of 1.493 percentage points; *p* < .001), while it does not reach statistical significance in East Germany (*p* = .374).

**Fig 3:**
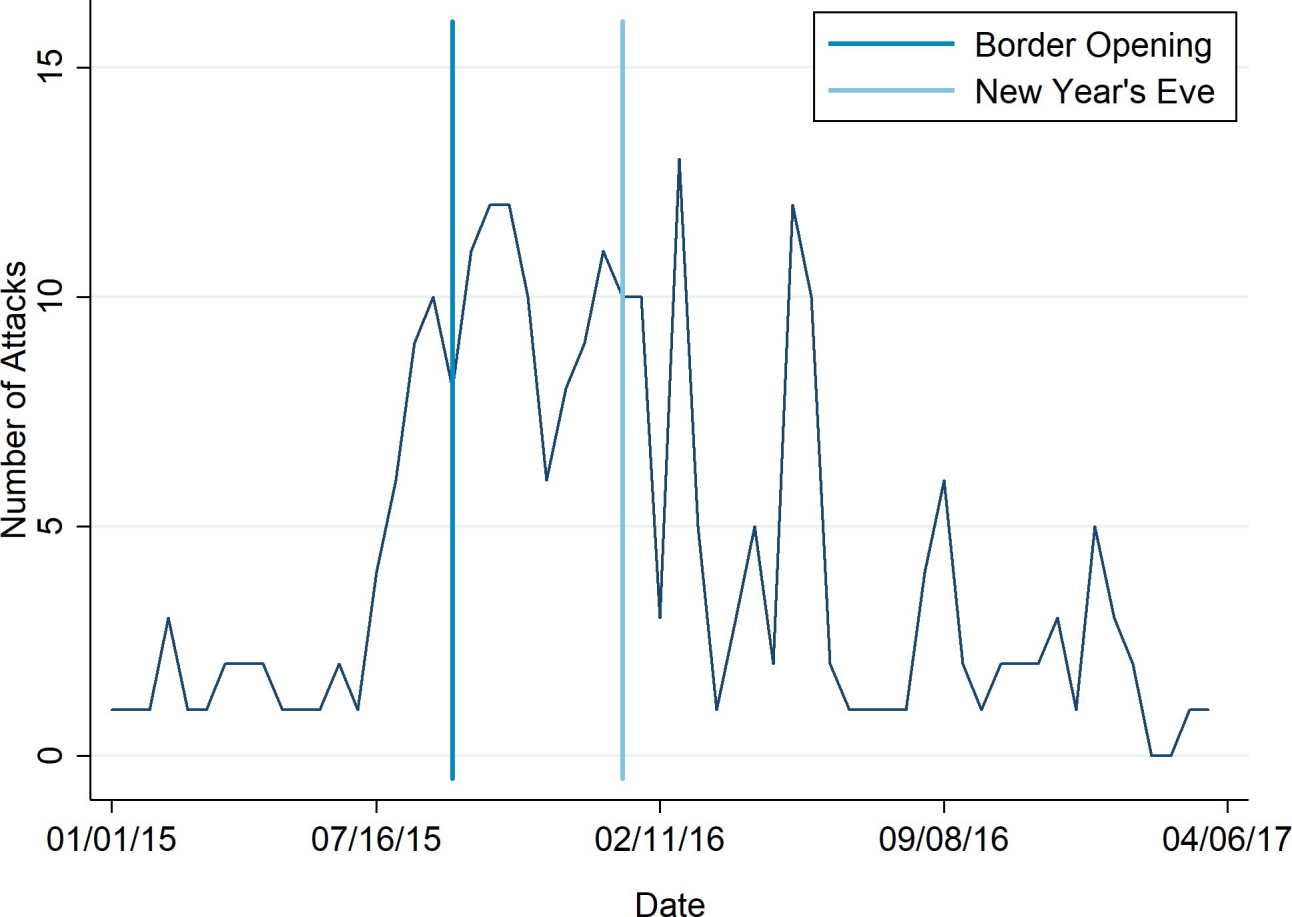
Number of arson attacks over time. The vertical lines represent the two key events: New Year’s Eve and the border opening.

Fig 3 illustrates the distributions of arson attacks over time in relation to the two salient threatening events. Arson attacks build up slowly before the border opening and reach a new peak following the event. This pattern is likely to be triggered by Merkel’s welcoming attitude towards refugees and the media coverage surrounding it. As mentioned above, the border opening was framed as both humanitarian help and a scenario of lost control over migration streams.

We will now consider the remaining time-invariant county-level factors (depicted in Fig 2) and the respective hypotheses related to these.

Regarding the *unemployment rate* (hypothesis e), our first proxy for economic threat, the models do not detect any influence at all (all coefficients fail to reach the usual level of statistical significance). In tendency, estimates for East Germany even reveal a negative trend: the higher unemployment rates, the lower the likelihood of an arson attack. In West Germany, we find a null effect with standard errors larger than coefficients.

From a theoretical perspective, the *proportion of foreigners* in the counties (hypothesis f) is ambivalent. As argued above, researchers have used this indicator to test economic threat as well as contact theory. The results from the models are crystal-clear: The higher the proportion of foreigners, the lower the likelihood of arson attacks. This is true for all models in Fig 2. To give one concrete example: In the general model for the whole of Germany, the average marginal effect is at -.099 percentage points, i.e., in counties with a proportion of foreigners of one fourth, the prevalence reduces by 2.5 percentage points in comparison to a county without any foreign inhabitants. By inspecting Fig 2B and 2C, one can conclude that this effect is similar in East and West-Germany. We tested further specifications for the main model to identify possible threshold effects, which might particularly play a role in counties with almost no foreigners (not reported, available on request). The results reported (in the first graph in Fig 2) stay stable and in line with contact theory.

Since the theoretical assumptions about economic threat might imply that the proportion of foreigners and the unemployment rate are intertwined, we tested for interaction effects (not reported, available on request). All different specifications (with continuous and categorical variables) did not point to any evidence of the unemployment rate affecting the spatial pattern of xenophobic violence.

As mentioned above, the *share of NPD voters*, the proxy for right-wing ideology (hypothesis g), is highly correlated with the dummy variable distinguishing East from West Germany. In the model for the whole of Germany, the share of NPD voters is associated with a higher likelihood of attacks. The average marginal effect is .227 percentage points (*p* = .040), i.e., a value of 5 percent share of NPD voters (which is close to the maximum value in the records) raises the prevalence of attacks by 1.135 percentage points. Thus, in counties with relatively many NPD voters, the prevalence rate approximately doubles (from .96 to 2.095). In separate models for East and West Germany, we see that the overall (positive) correlation is driven by the process in East Germany (average marginal effect at .155 percentage points, although clearly not significant with *p* = .436). In sum, the potential of right-wing extremists effects the diffusion of xenophobic violence, however other factors influence the pattern more strongly.

Last but not least, the control variable *size of population* clearly increases the likelihood of arson attacks: The more populated a county, the higher the prevalence. With each 100,000 inhabitants the likelihood increases by .180 percentage points (*p* < .001). This result stays stable when omitting Berlin from the data set (with a high number of arson attacks recorded).

With regard to *East and West Germany* (hypothesis h), the analyses show distinct patterns for some of our indicators, such as the share of NPD voters and fake news (see Fig 2). In other words, we see tendencies for interactions between region and indicator. However, when we include an East (vs West) dummy variable into the overall model for Germany and estimate its main effect (as sometimes done in other studies), no significant differences between East and West remain (see Table A model 6 in S3 Appendix).

To sum up, most of the considered variables are linked to the diffusion of arson attacks in Germany. The probability of an arson attack in a particular county increases with population size and the share of the right-wing extremists, while a higher share of foreigners clearly reduces the likelihood of arson attacks. The temporal proximity to Angela Merkel’s border opening and to the New Year’s Eve incidents triggered xenophobic events.

## Robustness checks

It is known from studies using time-dependent observational data that statistical models are sensitive to assumptions and to the specification of dependent and independent variables, specifically with regard to time-dependencies. We conducted several additional analyses to check for the robustness of our main results.

First, we ran an alternative model to the one presented in Fig 2, in which we additionally controlled for the proportion of asylum seekers in the counties in 2015 (see models 5 in S3 Appendix). The results indicate that the likelihood of arson attacks is not affected at all variation of asylum seekers registered in the individual counties. However, at closer inspection these data seemed to suffer from a drawback: There was a remarkable variance in asylum seekers by counties. This is astonishing because the official administrative strategy was to distribute refugees (more or less) equally across the counties. Our worry is that the reported numbers of the year 2015 might be biased due to administrative problems (such as double counts or the location of (first) registration facilities). For this reason, models have been checked using the statistics of the preceding year 2014 (results not shown but available upon request). Again, there is no measurable impact of the proportion of asylum seekers on the upcoming violence. It is not the difference in rising numbers of refugees on a local level that stimulates xenophobia. Second, we varied the lag structure of an attack from two weeks to four weeks, i.e. we relaxed the assumption that process time is only effective for one time-unit of two weeks after an attack. Results in the appendix highlight that all main findings stay stable (see Table A in S4 Appendix). Third, we changed the decay function of salient threatening events from a period of eight weeks to four, six, ten and twelve weeks. Again, results did not vary substantially (see Table B in S4 Appendix). The same holds for excluding the decay function from the model altogether and instead only using an indicator for the time period when the salient event occurred. Fourth, we also checked for second-next neighbor models as an alternative spatial structure. This specification performed worse than the models we reported, suggesting that contagion happens in a nearby environment rather than big geographical distances. Fifth, as already mentioned, we employed several models with interaction terms of proportion of foreigners and unemployment in order to grasp a moderated threat by migration. As reported, the negative main effect of exposure stayed highly significant–no matter in which economic situation. Note that the robustness checks beyond S4 Appendix are not reported, but available on request. We share our data compilation and syntax files for replication studies.

## Discussion and conclusion

Our analysis confirmed that xenophobic events, and more specifically arson attacks during the refugee crisis in Germany, are not only correlated with socio-economic characteristics such as population size, proportion of foreigners and party support for right-wing extremists. The focus and main contribution of our analysis is the fine-grained modeling of variables whose impact varies in time and space: previous attacks, local media coverage of attacks, fake news about refugees, and nation-wide salient events. What we clearly detected is that xenophobic violence also follows time- and space-specific patterns. Most importantly, arson attacks are not scattered randomly across German counties. It seems that a combination of an intolerant ideology (shared by a small but still visible minority), generally low exposure to foreigners, and reactions to salient threatening events and to fake news drove the process of violent behavior. Contradictory to a popular point of view, economic threat does not seem to be relevant at all.

When taking all covariates into account, the overall difference between both parts of Germany (which was visually depicted in a map of arson attacks in Fig 1) disappears. Nonetheless, there are distinct patterns for East and West Germany with regard to the impact of the other covariates: Strikingly, the effect sizes in East Germany are more pronounced, specifically for the negative coefficient for proportion of foreigners, the positive population size coefficient and the temporal proximity to keeping the border open (in September 2015). While previous attacks in geographical proximity do not drive arson attacks in East Germany, they do in the West. On the other hand, fake news about refugees only relate to violence in East Germany.

In sum, our analysis corroborates previous research about the political East-West cleavage, suggesting a greater detachment from political elites in East than in West Germany, who are perceived as responsible for the migration peak in 2015.

With regard to media influence on diffusion of violent behavior, we conclude that mainly salient events that received nation-wide media coverage are influential. Local newspaper coverage of (local) arson attacks–even in an aggressive tabloid such as BILD–does not boost the diffusion of violence. In different words, our approach of modeling local media coverage could not reveal an independent influence of local reports in addition to the effect that the sheer event of a nearby attack has. Comparing the impact of local media reports to the information about local fake news that we exploited, the latter are more likely to trigger the diffusion of violent attacks. This is specifically interesting because fake news by definition only affect the perception of citizens–they are not real by any means. Hard facts about economic differences across regions such as unemployment rates, however, do not impact the process under study at all.

What politics might learn from this is that the goal of an equal distribution of refugees to all counties is not a good idea–even if it seems to go along with the principle of sharing an equal burden. In the interest of a peaceful integration of refugees, political actors and public administration could use results from research to identify conditions that are more welcoming. An alternative distribution rule could consider local labor market conditions as another parameter. Along those lines, Bansak et al. [91] suggested to use algorithms to reach a better integration of refugees into local labor markets for the US and Switzerland.

Finally, let us discuss the ecological character of the analysis, which somewhat contradicts methodological individualism with its agent-centered models. We have examined correlations between macro phenomena or “social facts” as Durkheim would have called them [92]. We relate to the term insofar as the societal structures and norms that we describe in the context of violence against refugees might to a certain extent be perceived as an external force that exercises control over what are legitimate patterns of thinking and acting for individuals. Past arson attacks on refugee homes and their media coverage can contribute to the perception of a new social reality that in turn leads to observable socio-geographic diffusion patterns of violence. Durkheim’s suggestion that social facts should be explained merely by antecedent social phenomena ([92]: 193) is, however, not satisfying from the standpoint of methodological individualism, which would rather try to offer more thorough and comprehensive explanations by going down to the individual micro-level. The attentive reader will have noticed that despite the fact that our research question heavily draws on county-level correlations, we still use micro explanations to make our hypotheses plausible on a theoretical level. As a matter of fact, formalizing these potential underlying mechanisms would have required individual-level data on how news reception affects normative beliefs, or even information on the individuals that planned and carried out arson attacks. Going there would not only have been a different research project, it is also where big data approaches reach their limits and surveys, interviews or experiments have to take over.

## Supporting information

S1 Appendix Correlation matrix. (PDF)

S2 Appendix List of included regional editions of BILD. (PDF)

S3 Appendix Regression tables of main analysis. (PDF)

S4 Appendix Robustness checks. (PDF)

S1 File Replication material (stata code + data). (ZIP)
