# Supplementary material for: Media coverage, fake news, and the diffusion of xenophobic violence: A fine-grained county-level analysis of the geographic and temporal patterns of arson attacks during the German refugee crisis 2015–2017
Source: PLoS One. 2023 Jul 20;18(7):e0288645. doi: 10.1371/journal.pone.0288645 (PMC10358929; doi:10.1371/journal.pone.0288645)
Supplement: S1 Appendix — (PDF) [file pone.0288645.s001.pdf]

S1 Correlation Matrix

	Population	Unemployment	Foreigners	Asylum Seekers	NPD-Voters	Border Opening	New Year's Eve	Previous Attacks	Local Media	Fake News	Eastern Germany
Population	1.000										
Unemployment	0.152***	1.000									
Foreigners	0.292***	-0.038***	1.000								
Asylum Seekers	-0.006	0.090***	0.030***	1.000							
NPD-Voters	-0.091***	0.457***	-0.475***	0.021**	1.000						
Border Opening	-0.000	0.000	0.000	0.000	0.000	1.000					
New Year's Eve	-0.000	0.000	0.000	0.000	0.000	-0.069***	1.000				
Previous Attacks	0.075***	0.089***	-0.085***	-0.006	0.112***	0.083***	0.068***	1.000			
Local Media	0.020**	0.055***	-0.048***	-0.007	0.082***	0.109***	0.013	0.377***	1.000		
Fake News	0.035***	-0.002	-0.019**	0.000	0.040***	0.052***	0.192***	0.088***	0.037***	1.000	
Eastern Germany	0.010	0.494***	-0.505***	0.019**	0.813***	0.000	0.000	0.139***	0.079***	0.049***	1.000
