# Supplementary material for: Media coverage, fake news, and the diffusion of xenophobic violence: A fine-grained county-level analysis of the geographic and temporal patterns of arson attacks during the German refugee crisis 2015–2017
Source: PLoS One. 2023 Jul 20;18(7):e0288645. doi: 10.1371/journal.pone.0288645 (PMC10358929; doi:10.1371/journal.pone.0288645)
Supplement: S2 Appendix — (PDF) [file pone.0288645.s002.pdf]

S2 List of included regional editions of BILD

- Bild Berlin-Brandenburg
- Bild Bund
- Bild Dresden
- Bild Düsseldorf
- Bild Frankfurt
- Bild Halle
- Bild Hamburg
- Bild Hannover
- Bild Köln
- Bild Leipzig
- Bild Magdeburg
- Bild Mainz-Wiesbaden
- Bild Mecklenburg-Vorpommern
- Bild München
- Bild Nürnberg
- Bild Rhein-Neckar
- Bild Ruhrgebiet
- Bild Saarland
- Bild Sachsen-Anhalt
- Bild Stuttgart
- Bild Thüringen
