# Supplementary material for: Media coverage, fake news, and the diffusion of xenophobic violence: A fine-grained county-level analysis of the geographic and temporal patterns of arson attacks during the German refugee crisis 2015–2017
Source: PLoS One. 2023 Jul 20;18(7):e0288645. doi: 10.1371/journal.pone.0288645 (PMC10358929; doi:10.1371/journal.pone.0288645)
Supplement: S3 Appendix — (PDF) [file pone.0288645.s003.pdf]

S3 Regression Tables of Main Analysis

The following tables provide the average marginal effects (AME) from the discrete event history model, their standard errors, their p values, and indicate statistically significant results. The coefficients indicate the average changes in the probability of an arson attack if the predictor variable increases by one unit. Tables S3.1 refers to Germany as a whole, Table shows data for West Germany S3.2 and Table S3.3 for East Germany.

Each table contains several model specifications. All of the models include the time-constant variables for the individual counties (population, unemployment, foreigners NPD voters), and the two salient threatening events with nation-wide media coverage (border opening and New Year's Eve).

Model 1 additionally contains the lag variable of previous attacks, **Models 2** shows the local media coverage of arson attacks and **Model 3** the number of fake news. Model 2 and 3 do not contain the lagged number of previous attacks – in order to detect any substantive problems with multi-collinearity.

Model 4 gives the coefficients of a complete model for all variables. This is also what we show in Figure 2.

Model 5 additionally tests the possible impact of the number of asylum seekers per county. We use the numbers from 2015 – and for robustness checks also the numbers from 2014 (i.e. from before the observation time).

Model 6 contains a dummy variable indicating counties in East Germany.

Since the main effects of all variables considered are stable across the models, we only discuss the results from the complete model 4 in the main text of the paper.

S3 Table A: Average marginal effects for Germany

	(1)<br>Attacks	(2)<br>Media	(3)<br>Fake News	(4)<br>Complete	(5)<br>Complete + AS	(6)<br>Complete + AS + E/W
Arson Attack (y/n)						
Population	0.182***	0.189***	0.187***	0.181***	0.180***	0.179***
	(0.027)	(0.028)	(0.028)	(0.027)	(0.027)	(0.028)
	[0.000]	[0.000]	[0.000]	[0.000]	[0.000]	[0.000]
Unemployment	-0.009	-0.007	-0.006	-0.008	-0.006	-0.007
	(0.034)	(0.034)	(0.034)	(0.034)	(0.034)	(0.036)
	[0.790]	[0.841]	[0.868]	[0.820]	[0.871]	[0.852]
Foreigners	-0.099***	-0.102***	-0.102***	-0.099***	-0.098***	-0.097***
	(0.026)	(0.026)	(0.026)	(0.026)	(0.026)	(0.028)
	[0.000]	[0.000]	[0.000]	[0.000]	[0.000]	[0.001]
Asylum Seekers					-0.067	-0.067
					(0.132)	(0.132)
					[0.611]	[0.612]
NPD-Voters	0.233*	0.241*	0.237*	0.230*	0.227*	0.217
	(0.110)	(0.112)	(0.112)	(0.110)	(0.111)	(0.149)
	[0.035]	[0.032]	[0.034]	[0.037]	[0.040]	[0.145]
Border Opening	1.435***	1.486***	1.467***	1.434***	1.434***	1.434***
	(0.247)	(0.248)	(0.247)	(0.250)	(0.250)	(0.250)
	[0.000]	[0.000]	[0.000]	[0.000]	[0.000]	[0.000]
New Year's Eve	1.136***	1.182***	1.138***	1.094***	1.094***	1.094***
	(0.271)	(0.271)	(0.274)	(0.275)	(0.275)	(0.275)
	[0.000]	[0.000]	[0.000]	[0.000]	[0.000]	[0.000]
Previous Attacks	0.287**			0.307**	0.307**	0.306**
	(0.105)			(0.110)	(0.110)	(0.110)
	[0.006]			[0.005]	[0.005]	[0.005]
Local Media		0.100		-0.300	-0.303	-0.302
		(0.409)		(0.436)	(0.436)	(0.436)
		[0.807]		[0.491]	[0.487]	[0.489]
Fake News			0.081	0.071	0.071	0.071
			(0.064)	(0.069)	(0.069)	(0.069)
			[0.210]	[0.302]	[0.301]	[0.302]
Eastern Germany						0.040
						(0.391)
						[0.919]
Observations	23316	23316	23316	23316	23316	23316
chi2	134.985	122.631	124.660	136.974	137.050	137.083
p	0.000	0.000	0.000	0.000	0.000	0.000

Marginal effects
Standard errors in parentheses
p-values in square brackets
* p<0.05, ** p<0.01, *** p<0.001

S3 Table B: Average Marginal Effects for West Germany

	(1) Attacks	(2) Media	(3) Fake News	(4) Complete	(5) Complete + AS
Arson Attack (y/n)					
Population	0.248***	0.257***	0.263***	0.250***	0.250***
	(0.047)	(0.048)	(0.049)	(0.048)	(0.048)
	[0.000]	[0.000]	[0.000]	[0.000]	[0.000]
Unemployment	0.016	0.021	0.020	0.012	0.012
	(0.042)	(0.043)	(0.043)	(0.043)	(0.043)
	[0.697]	[0.619]	[0.637]	[0.775]	[0.788]
Foreigners	-0.100***	-0.104***	-0.104***	-0.099***	-0.099***
	(0.029)	(0.029)	(0.029)	(0.029)	(0.029)
	[0.000]	[0.000]	[0.000]	[0.001]	[0.001]
Asylum Seekers					0.014
					(0.139)
					[0.917]
NPD-Voters	-0.247	-0.250	-0.248	-0.259	-0.258
	(0.284)	(0.286)	(0.288)	(0.285)	(0.285)
	[0.385]	[0.381]	[0.389]	[0.362]	[0.366]
Border Opening	1.353***	1.362***	1.455***	1.327***	1.327***
	(0.327)	(0.334)	(0.323)	(0.336)	(0.336)
	[0.000]	[0.000]	[0.000]	[0.000]	[0.000]
New Year's Eve	1.319***	1.469***	1.629***	1.493***	1.493***
	(0.334)	(0.320)	(0.335)	(0.346)	(0.346)
	[0.000]	[0.000]	[0.000]	[0.000]	[0.000]
Previous Attacks	0.452**			0.467**	0.466**
	(0.169)			(0.181)	(0.181)
	[0.008]			[0.010]	[0.010]
Local Media		0.700		0.293	0.294
		(0.555)		(0.580)	(0.580)
		[0.207]		[0.613]	[0.612]
Fake News			-0.246	-0.321	-0.321
			(0.226)	(0.241)	(0.241)
			[0.277]	[0.182]	[0.182]
Observations	18850	18850	18850	18850	18850
chi2	83.444	73.376	71.517	86.068	86.115
p	0.000	0.000	0.000	0.000	0.000

Marginal effects
Standard errors in parentheses
p-values in square brackets
* p<0.05, ** p<0.01, *** p<0.001

S3 Table C: Average Marginal Effects for East Germany

	(1) Attacks	(2) Media	(3) Fake News	(4) Complete	(5) Complete + AS
Arson Attack (y/n)					
Population	0.239***	0.245***	0.239***	0.236***	0.232***
	(0.053)	(0.054)	(0.053)	(0.053)	(0.053)
	[0.000]	[0.000]	[0.000]	[0.000]	[0.000]
Unemployment	-0.071	-0.072	-0.066	-0.062	-0.057
	(0.067)	(0.068)	(0.067)	(0.067)	(0.069)
	[0.289]	[0.293]	[0.328]	[0.351]	[0.410]
Foreigners	-0.362**	-0.371**	-0.367**	-0.364**	-0.350**
	(0.135)	(0.136)	(0.135)	(0.135)	(0.136)
	[0.007]	[0.007]	[0.007]	[0.007]	[0.010]
Asylum Seekers					-0.238
					(0.371)
					[0.522]
NPD-Voters	0.185	0.187	0.153	0.159	0.155
	(0.197)	(0.200)	(0.198)	(0.197)	(0.199)
	[0.348]	[0.351]	[0.440]	[0.419]	[0.436]
Border Opening	1.525***	1.589***	1.226**	1.236**	1.236**
	(0.381)	(0.380)	(0.418)	(0.419)	(0.419)
	[0.000]	[0.000]	[0.003]	[0.003]	[0.003]
New Year's Eve	0.592	0.575	0.432	0.454	0.455
	(0.506)	(0.506)	(0.515)	(0.512)	(0.512)
	[0.242]	[0.255]	[0.401]	[0.375]	[0.374]
Previous Attacks	0.168			0.207	0.206
	(0.137)			(0.143)	(0.143)
	[0.218]			[0.147]	[0.149]
Local Media		-0.427		-0.821	-0.822
		(0.611)		(0.659)	(0.659)
		[0.485]		[0.213]	[0.212]
Fake News			0.335**	0.329**	0.327**
			(0.119)	(0.120)	(0.121)
			[0.005]	[0.006]	[0.007]
Observations	4466	4466	4466	4466	4466
chi2	45.004	42.986	52.236	55.233	55.178
p	0.000	0.000	0.000	0.000	0.000

Marginal effects
Standard errors in parentheses
p-values in square brackets
* p<0.05, ** p<0.01, *** p<0.001
