# Supplementary material for: Media coverage, fake news, and the diffusion of xenophobic violence: A fine-grained county-level analysis of the geographic and temporal patterns of arson attacks during the German refugee crisis 2015–2017
Source: PLoS One. 2023 Jul 20;18(7):e0288645. doi: 10.1371/journal.pone.0288645 (PMC10358929; doi:10.1371/journal.pone.0288645)
Supplement: S4 Appendix — (PDF) [file pone.0288645.s004.pdf]

S4 Robustness Checks

S4 Table A: Robustness check by lagging two time periods, i.e., using the number of events (attacks, media reports, fake news) in the previous four weeks.

	(1) Germany	(2) West Germany	(3) East Germany
Arson Attack (y/n)			
Population	0.170***	0.243***	0.219***
	(0.027)	(0.047)	(0.052)
	[0.000]	[0.000]	[0.000]
Unemployment	-0.007	0.011	-0.056
	(0.033)	(0.043)	(0.067)
	[0.824]	[0.793]	[0.401]
Foreigners	-0.095***	-0.097***	-0.333*
	(0.026)	(0.029)	(0.134)
	[0.000]	[0.001]	[0.013]
Asylum Seekers	-0.063	0.016	-0.216
	(0.130)	(0.137)	(0.351)
	[0.628]	[0.906]	[0.538]
NPD-Voters	0.209	-0.268	0.140
	(0.109)	(0.283)	(0.195)
	[0.055]	[0.344]	[0.472]
Border Opening	1.269***	1.229***	1.033*
	(0.257)	(0.342)	(0.427)
	[0.000]	[0.000]	[0.015]
New Year's Eve	1.009***	1.370***	0.394
	(0.278)	(0.354)	(0.516)
	[0.000]	[0.000]	[0.445]
Previous Attacks (4 Weeks)	0.213**	0.310*	0.145
	(0.072)	(0.133)	(0.090)
	[0.003]	[0.020]	[0.108]
Local Media (4 Weeks)	0.329	0.472	0.166
	(0.281)	(0.437)	(0.365)
	[0.242]	[0.280]	[0.649]
Fake News (4 Weeks)	0.074	-0.121	0.250**
	(0.047)	(0.130)	(0.082)
	[0.117]	[0.354]	[0.002]
Observations	22914	18525	4389
chi2	149.776	87.116	60.544
p	0.000	0.000	0.000

Marginal effects
Standard errors in parentheses
p-values in square brackets
* p<0.05, ** p<0.01, *** p<0.001

S4 Table B: Robustness check by changing the decay function, i.e., changing the influence of the key events (New Year's Eve and the border opening) to range between four and twelve weeks.

	(1)<br>4 Week<br>Decay	(2)<br>6 Week<br>Decay	(3)<br>8 Week<br>Decay	(4)<br>10 Week<br>Decay	(5)<br>12 Week<br>Decay
Arson Attack (y/n)					
Population	0.178***	0.248***	0.250***	0.251***	0.250***
	(0.027)	(0.047)	(0.048)	(0.048)	(0.048)
	[0.000]	[0.000]	[0.000]	[0.000]	[0.000]
Unemployment	-0.006	0.012	0.012	0.011	0.012
	(0.034)	(0.043)	(0.043)	(0.043)	(0.043)
	[0.867]	[0.772]	[0.788]	[0.791]	[0.787]
Foreigners	-0.097***	-0.099***	-0.099***	-0.099***	-0.099***
	(0.026)	(0.029)	(0.029)	(0.029)	(0.029)
	[0.000]	[0.001]	[0.001]	[0.001]	[0.001]
Asylum Seekers	-0.067	0.014	0.014	0.015	0.015
	(0.132)	(0.138)	(0.139)	(0.139)	(0.138)
	[0.611]	[0.918]	[0.917]	[0.916]	[0.916]
NPD-Voters	0.225*	-0.258	-0.258	-0.258	-0.257
	(0.110)	(0.285)	(0.285)	(0.285)	(0.285)
	[0.041]	[0.364]	[0.366]	[0.367]	[0.367]
Border Opening (4-12W Decay)	1.019***	1.165***	1.327***	1.410***	1.383***
	(0.296)	(0.353)	(0.336)	(0.330)	(0.332)
	[0.001]	[0.001]	[0.000]	[0.000]	[0.000]
New Year's Eve (4-12W Decay)	0.842**	1.184**	1.493***	1.535***	1.469***
	(0.311)	(0.380)	(0.346)	(0.343)	(0.348)
	[0.007]	[0.002]	[0.000]	[0.000]	[0.000]
Previous Attacks	0.322**	0.497**	0.466**	0.449*	0.452*
	(0.109)	(0.181)	(0.181)	(0.182)	(0.183)
	[0.003]	[0.006]	[0.010]	[0.014]	[0.013]
Local Media	-0.146	0.324	0.294	0.269	0.298
	(0.434)	(0.581)	(0.580)	(0.580)	(0.579)
	[0.736]	[0.578]	[0.612]	[0.643]	[0.606]
Fake News	0.098	-0.254	-0.321	-0.334	-0.318
	(0.058)	(0.236)	(0.241)	(0.241)	(0.240)
	[0.090]	[0.282]	[0.182]	[0.165]	[0.185]
Observations	23316	18850	18850	18850	18850
chi2	115.041	76.321	86.115	88.003	85.122
p	0.000	0.000	0.000	0.000	0.000

Marginal effects
Standard errors in parentheses
p-values in square brackets
* p<0.05, ** p<0.01, *** p<0.001
